# Repetitive transcranial magnetic stimulation alleviates glial activation through suppressing HMGB1/TLR4 pathway in a rat model of Parkinson’s disease

**DOI:** 10.1007/s11033-023-08561-8

**Published:** 2023-06-16

**Authors:** Chao Han, Xue Zhang, Kaixin Dou, Weichao Yao, Minyi Yao, Qi Wan, Anmu Xie

**Affiliations:** 1 Department of Neurology, Affiliated Hospital of Qingdao University, No.16 Jiangsu Road, Qingdao, 266003 Shandong Province People’s Republic of China; 2 Department of Physical Medicine and Rehabilitation, Affiliated Hospital of Qingdao University, Qingdao, People’s Republic of China; 3 Institute of Neuroregeneration & Neurorehabilitation, Qingdao University, 308 Ningxia Street, Qingdao, 266071 Shandong Province People’s Republic of China; 4 Department of Neurology, Affiliated Qingdao Central Hospital of Qingdao University, Qingdao, People’s Republic of China

**Keywords:** Parkinson's disease, Neuroinflammation, HMGB1, Astrocyte, Microglia, rTMS

## Abstract

**Background:**

Repetitive transcranial magnetic stimulation (rTMS) has been demonstrated to be effective in Parkinson’s disease (PD), but whether rTMS treatment has a relieving effect on neuroinflammation remains to be investigated. In this article, we explored the effects of rTMS on forelimb use asymmetry and neuroinflammation-related mechanisms in a 6-hydroxydopamine (6-OHDA)-induced PD rat model.

**Methods and results:**

Rats in the 6-OHDA+rTMS group received 10 Hz rTMS daily for 4 weeks. Behavioral tests (the cylinder test) were performed at the 3rd and 7th weeks after the operation. Astrocyte and microglia activation and protein levels of tyrosine hydroxylase(TH), high-mobility group box 1(HMGB1) and toll-like receptors 4(TLR4) were investigated by immunohistochemistry and Western blot analyses, respectively. After 4 weeks of treatment, forelimb use asymmetry was ameliorated in the 6-OHDA+rTMS group. Consistent with the behavioral tests, rTMS increased TH in the substantia nigra (SN) and the striatum of PD rats. High glial activation and HMGB1/TLR4 expression in the SN and the striatum were observed in the 6-OHDA group, while rTMS alleviated these changes.

**Conclusions:**

This study showed that rTMS might be a promising method for alleviating neuroinflammation in PD rat models, and the effects might be mediated through the downregulation of the HMGB1/TLR4 pathway.

## Introduction

Parkinson’s disease (PD) is a complex and common neurodegenerative disorder with selective neuronal loss in the substantia nigra (SN) [1]. PD has complicated causes that have still not been fully elucidated. Previous studies have reported that neuroinflammation plays an important role in PD [2, 3]. It has been shown that the neuroinflammatory response mediated by glial cells is involved in the process of occurrence and development of PD [2, 4]. Neuroinflammation refers to the inflammatory process in the central nervous system caused by molecules released by immune cells in the brain and/or derived from the blood [5]. In the process of neuroinflammation, increased oxidative stress and glial cell activation may occur [6]. Activated astrocytes and microglial cells release proinflammatory and neurotoxic factors that can exacerbate brain damage [7]. The activated immune system may contribute to the repair of damaged tissues, but when it becomes dysregulated and maladaptive, disease is initiated [5]. Therefore, inflammatory pathways may be important treatment targets for PD. Studies have shown that repetitive transcranial magnetic stimulation (rTMS) may alleviate PD animal model symptoms [8] and have beneficial effects in PD patients [9, 10]. The underlying mechanisms of rTMS treatment are still not clear. Its anti-neuroinflammation activity and other relevant mechanisms require further study.

High-mobility group box 1/toll-like receptor 4 (HMGB1/TLR4) is considered an important inflammatory signaling pathway [11]. HMGB1 is a nuclear protein with high electrophoretic mobility that exists in all types of cells [12]. It regulates inflammation and the immune response after being released from damaged cells [12]. The two main receptors of HMGB1 are TLRs and receptor for advanced glycation end products (RAGE) [12]. HMGB1 has also been reported to mediate dopaminergic neuronal loss in PD animal models [12, 13]. The up- or downregulation of HMGB1/TLR4 pathway expression is closely associated with the development of PD and therapy effectiveness [14]. Unilateral intrastriatal 6-hydroxydopamine (6-OHDA) infusion in rats is often used to mimic the pathological features of PD in humans [15]. In this article, we focused on investigating the effects of rTMS on forelimb use asymmetry and neuroinflammation-related mechanisms in a 6-OHDA-induced PD rat model.

## Materials and methods

### Animals and reagents

Specific pathogen-free Sprague-Dawley male rats (age, 6 w; weight, 200–220 g) were purchased from Sibeifu (Beijing, China) Biotechnology Co., Ltd (license No. SCXK2019-0010). The rats were reared in clean cages at 22.0 ± 2.0 °C room temperature and 55 ± 10% relative humidity under a 12 h light and 12 h dark cycle. The animals were randomly divided into the following three groups: unilateral intrastriatal infusion of 6-OHDA hydrochloride (dissolved in 0.9% normal saline containing 0.02% ascorbic acid) followed by sham rTMS treatment (6-OHDA group), unilateral intrastriatal infusion of 6-OHDA hydrochloride (dissolved in normal saline containing ascorbic acid) followed by rTMS treatment (6-OHDA+rTMS group), and unilateral intrastriatal infusion with normal saline containing ascorbic acid (control group).

### Animal surgery

Rats in the 6-OHDA group and 6-OHDA+rTMS group were pretreated with desipramine (12.5 mg/kg, Med Chem Express, USA) 30 min before the operation. Isoflurane-anaesthetized rats were firmly fixed on a stereotaxic frame (Reward, China). 6-OHDA hydrochloride (20 µg/4 µl, Sigma, USA) dissolved in normal saline containing ascorbic acid (Sigma) was administered into the right striatum at 0.5 µl/min using a 10 µl syringe (Hamilton, Switzerland) at the following coordinates: anteroposterior, 0.7 mm; mediolateral, − 2.6 mm; dorsoventral, − 4.5 mm as previously described [15]. After remaining in place for 5 min, the inserted needle was slowly withdrawn (at 1 mm/min). At the 3rd week after 6-OHDA injection, apomorphine (0.5 mg/kg, dissolved in normal saline containing ascorbic acid, Sigma) was administered intraperitoneally [15]. Rats that showed continuous rotation > 2 turns/min towards the contralateral side relative to the injection side after the apomorphine injection were used in further experiments. Rats in the control group were administered 4 µl normal saline containing ascorbic acid into the right striatum using the same method.

### rTMS

The rats were restricted with a plastic holder when receiving rTMS treatment. A round coil (64-mm diameter) (Y064, YIRUIDE, Wuhan, China) was set parallel to the skull of the rats and kept 1 cm away from the rats head as described previously [8]. The parameters were set as follows: 10 Hz, approximately 1 Tesla magnetic field [8], 1 s stimulation, 9 s interval, 20 min treatment daily for 4 weeks. Rats in the 6-OHDA group were administered the same rTMS protocol, but the coil positioning was perpendicular to the skull of the rats.

### Behavioral tests

The cylinder test, which was used for evaluating forelimb use asymmetry, was performed at the 3rd and 7th weeks after the operation (test 1 and test 2, respectively). The rats were put into a transparent plexiglass cylinder (20 × 30 cm), and a mirror was placed behind the cylinder [16]. The rats were observed for 5 min, and the number of times the rat forelimbs contacted the cylinder wall was recorded. The final data were calculated by the equation [(ipsilateral (right) forepaw + 0.5 both paws)/(ipsilateral paw (right) + contralateral paw (left) + both paws)] * 100% [16].

### Immunohistochemistry staining

Glial fibrillary acidic protein (GFAP) and Ionized calcium-binding adapter molecule 1(Iba-1) are generally used as markers of glial cells (astrocytes and microglia). One day after the second behavioral test, 5 rats from each group were selected randomly for immunohistochemistry staining. The anaesthetized animals were intracardially perfused with 0.9% normal saline and 4% paraformaldehyde (Solarbio, China). The rat brains were rapidly dissected out and immersed in paraformaldehyde at 4 °C. After dehydration and clearing, the tissues were embedded in paraffin and then cut into 5-µm coronal slices. Then, the slices were deparaffinized and hydrated. Afterwards, the slices were unmasked in 0.01 M citric acid buffer and incubated with 3% hydrogen peroxide in the dark followed by primary antibodies (anti-GFAP 1:1000, Abcam; anti-Iba-1 1:1000, Wako, Japan; anti- tyrosine hydroxylase (TH) 1:1000, Millipore, USA) overnight at 4 °C. The corresponding secondary antibodies were then added, followed by staining with diaminobenzidine (DAB) until the staining was deemed satisfactory. Hematoxylin staining of the nuclei was carried out, followed by graded ethanol dehydration and fixation in transparent neutral gum. Images were obtained with an Olympus BX53 microscope. ImageJ software was used to analyse positively stained areas.

### Western blot analysis

**Fig. 1:**
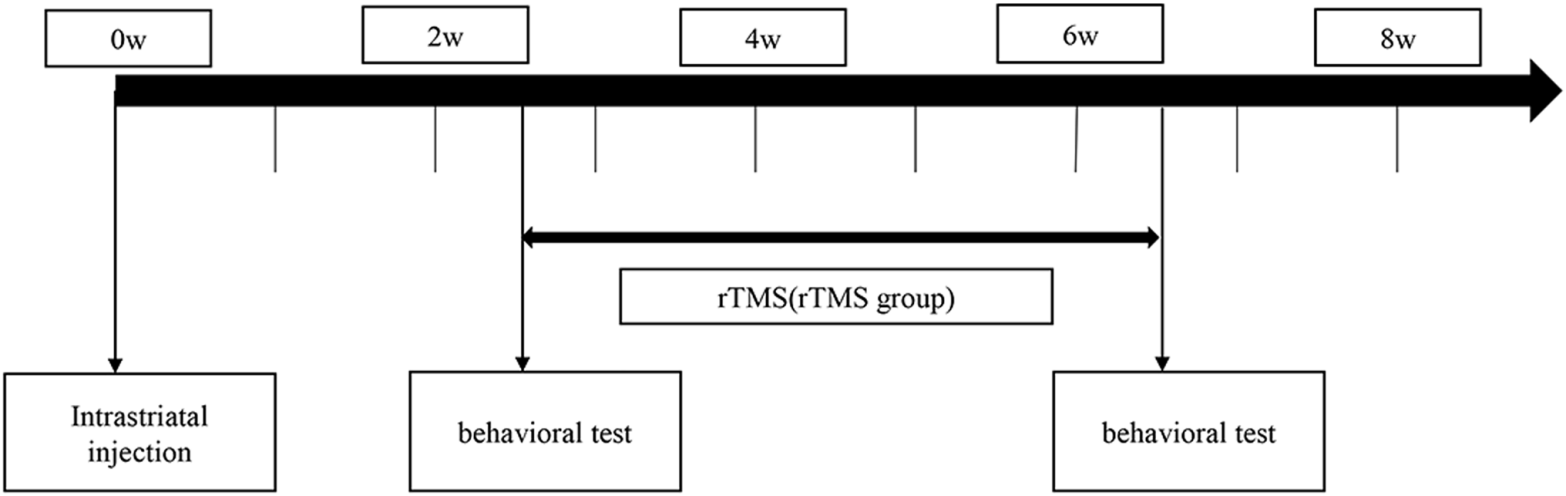
Experimental design. Intrastriatal injection of 6-OHDA hydrochloride dissolved in normal saline containing ascorbic acid (6-OHDA group and 6-OHDA+rTMS group) or normal saline containing ascorbic acid (control group) was carried out at 0 w. Behavioral tests (the cylinder test) were performed at the 3rd and 7th weeks after the operation. Rats in the 6-OHDA+rTMS group received rTMS daily for 4 weeks (3rd week–7th week). Rats were sacrificed after the second behavioral test

Rat striatum and SN tissues were lysed in RIPA lysis buffer (Applygen, China) containing protease inhibitor and phosphatase inhibitor (Solarbio) on ice. Protein concentrations in the striatum and SN were determined using a BCA assay (Thermo Fisher Scientific, USA). Protein samples were separated by 10% SDS-PAGE and then transferred to PVDF membranes(Millipore) at 320 mA for 1 h. The membranes were blocked with 5% skim milk for 2 h. The samples were then incubated with the corresponding primary antibodies (anti-HMGB1 1:10000, Abcam, UK; anti-β-actin 1:4000, Proteintech, China; anti-TLR4 1:1000, Santa Cruz, USA; anti- TH 1:1000, Millipore) overnight at 4 °C. The next day, the membranes were incubated with the corresponding secondary antibodies (1:10,000, Kerui Biotechnology, China) for 1 h after washing. A chemiluminescence (ECL) reagent (Millipore) were added to visualize the bands after washing. The optical densities of the bands were calculated using ImageJ software (Fig. 1).

### Statistics

Statistical analysis was carried out using statistical software (GraphPad Prism 8, GraphPad Software Inc., USA). The apomorphine-induced rotational test of the 6-OHDA group and the 6-OHDA+rTMS group was analysed by *t* test. The comparisons of other results were analysed by analysis of variance (ANOVA) followed by Sidak’s or Tukey’s post hoc comparisons. Statistical differences were considered when P < 0.05.

## Results

### rTMS treatment improved motor function in PD rats

For the apomorphine-induced rotation test, no significant difference was observed between the 6-OHDA group (4.960 ± 1.854 r/min) and the 6-OHDA+rTMS group (4.830 ± 1.779 r/min) (P > 0.05).

**Fig. 2:**
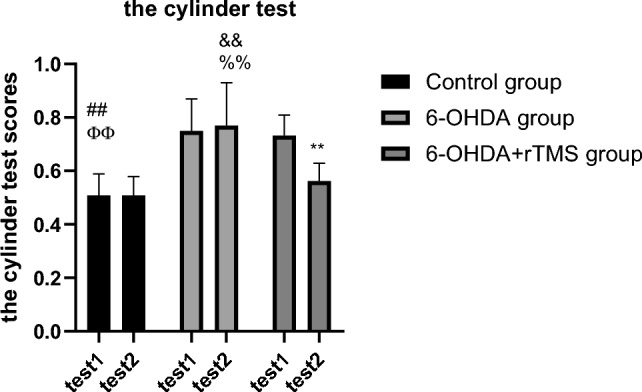
The cylinder test scores. ∗∗P < 0.01 vs. test1, ##P < 0.01 vs. 6-OHDA group, ФФP < 0.01 vs. 6-OHDA+rTMS group, &&P < 0.01 vs. control group, %%P < 0.01 vs. 6-OHDA+rTMS group. Test 1: The cylinder test was performed at the 3rd week after the operation. Test 2: The cylinder test was performed at the 7th week after the operation

Compared with test 1, the cylinder test scores decreased significantly after 4 weeks only in the 6-OHDA+rTMS group (P < 0.01) (Fig. 2). When comparing among the 3 groups, the cylinder test scores of both the 6-OHDA and 6-OHDA+rTMS groups were increased at the 3rd week after operation compared with those in the control group (P < 0.01), indicating that 6-OHDA-lesioned rats used the ipsilateral forelimb more than the contralateral forelimb. There were no significant differences in the cylinder test scores between the 6-OHDA and 6-OHDA+rTMS groups (P > 0.05). After 4 weeks, the cylinder test score improvements were more significant in the 6-OHDA+rTMS group than in the 6-OHDA group (P < 0.01). This suggested that rTMS treatment improved motor function in PD rats (Fig. 2).

### rTMS improved TH expression levels in PD rats

**Fig. 3:**
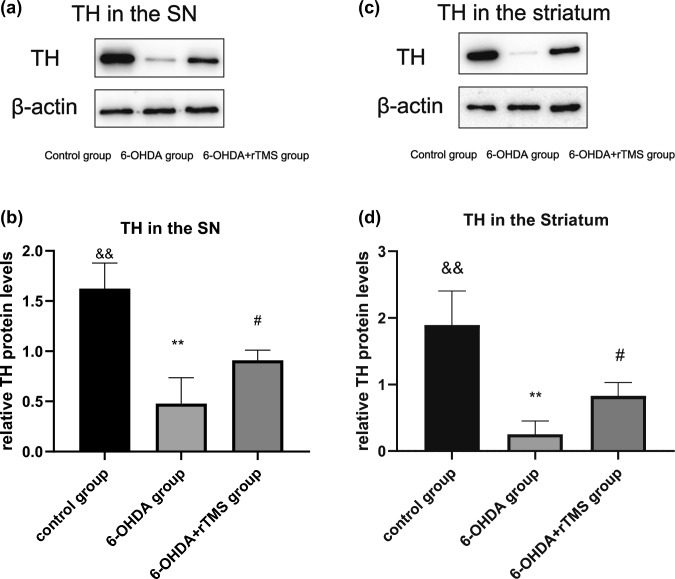
TH expression assessed by Western blot analysis (n = 5). **a** Typical immunoblots in the substantia nigra. **b** Mean ratio of TH densitometry density data in relation to β-actin in the substantia nigra. **c** Typical immunoblots in the striatum. **d** Mean ratio of TH densitometry density data in relation to β-actin in the striatum. ∗∗P < 0.01 vs. control group, #P < 0.05 vs. 6-OHDA group, &&P < 0.01 vs. 6-OHDA+rTMS group

**Fig. 4:**
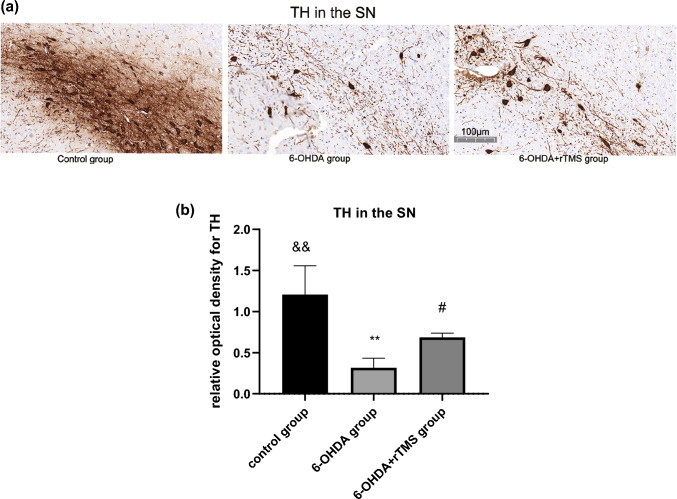
**a** Digital images of coronal sections of the SN stained for TH. **b** Relative optical density for TH in the SN. ∗∗P < 0.01 vs. control group, #P < 0.05 vs. 6-OHDA group, &&P < 0.01 vs. 6-OHDA+rTMS group

When compared with the control group, administration of 6-OHDA reduced the expression of TH in the SN and the striatum in both the 6-OHDA and 6-OHDA+rTMS groups (P < 0.01). However, rTMS significantly improved the TH expression level compared with that in the 6-OHDA group (P < 0.05) (Figs. 3 and 4).

### rTMS ameliorated astrocyte and microglia activation in PD rats

**Fig. 5:**
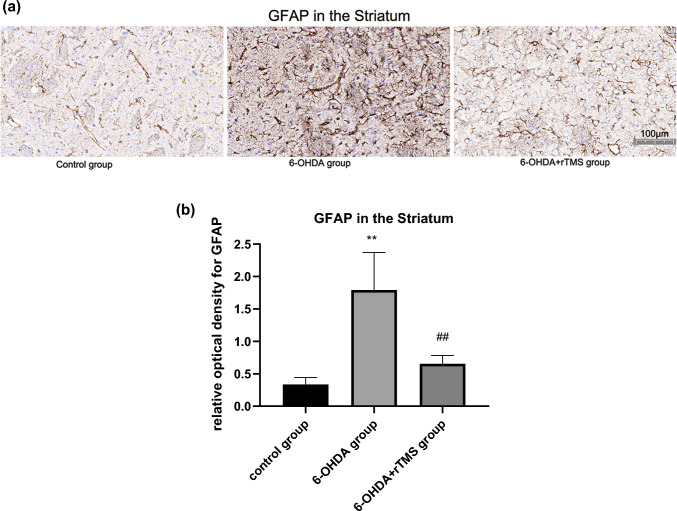
**a** Digital images of coronal sections of the striatum stained for GFAP. **b** Relative optical density for GFAP in the striatum. ∗∗P < 0.01 vs. control group, ##P < 0.01 vs. 6-OHDA group

**Fig. 6:**
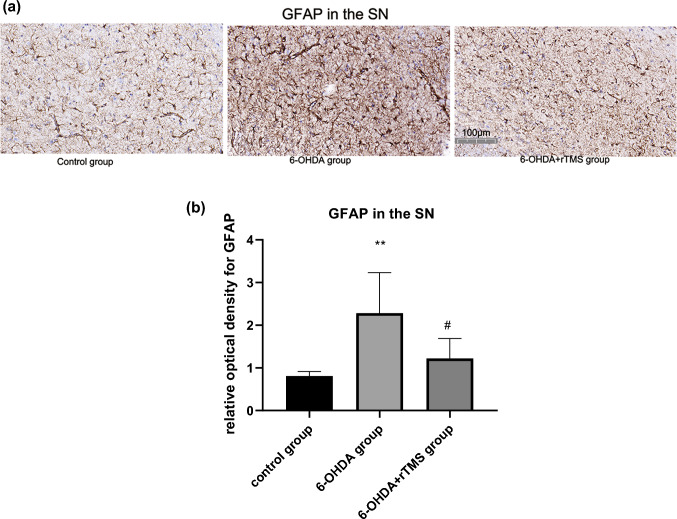
**a** Digital images of coronal sections of the SN stained for GFAP. **b** Relative optical density for GFAP in the SN. ∗∗P < 0.01 vs. control group, #P < 0.05 vs. 6-OHDA group

**Fig. 7:**
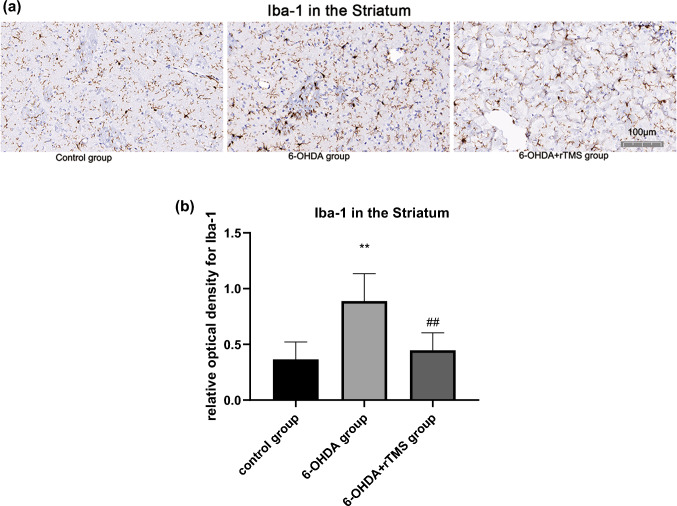
**a** Digital images of coronal sections of the striatum stained for Iba-1. **b** Relative optical density for Iba-1 in the striatum. ∗∗P < 0.01 vs. control group, ##P < 0.01 vs. 6-OHDA group

**Fig. 8:**
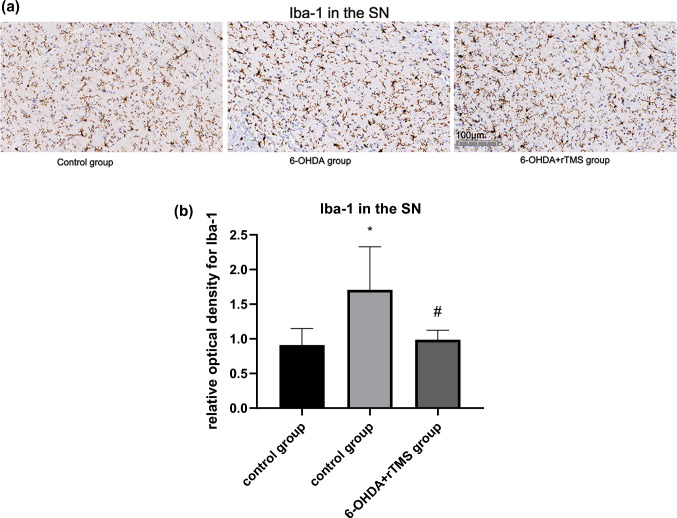
**a** Digital images of coronal sections of the SN stained for Iba-1. **b** Relative optical density for Iba-1 in the SN. ∗P < 0.05 vs. control group, #P < 0.05 vs. 6-OHDA group

Astrocyte activation in the ipsilateral striatum and SN of the 6-OHDA group was significantly higher than that of the control group (P < 0.01). However, GFAP expression in the 6-OHDA+rTMS group were significantly decreased compared with that in the 6-OHDA group (P < 0.01 and P < 0.05, respectively) (Figs. 5 and 6). For microglia activation in the striatum and SN, high microglia activation was observed in the 6-OHDA group (P < 0.01 and P < 0.05, respectively), while rTMS alleviated these changes (P < 0.01 and P < 0.05, respectively) (Figs. 7 and 8).

### rTMS decreased HMGB1/TLR4 levels in PD rats

**Fig. 9:**
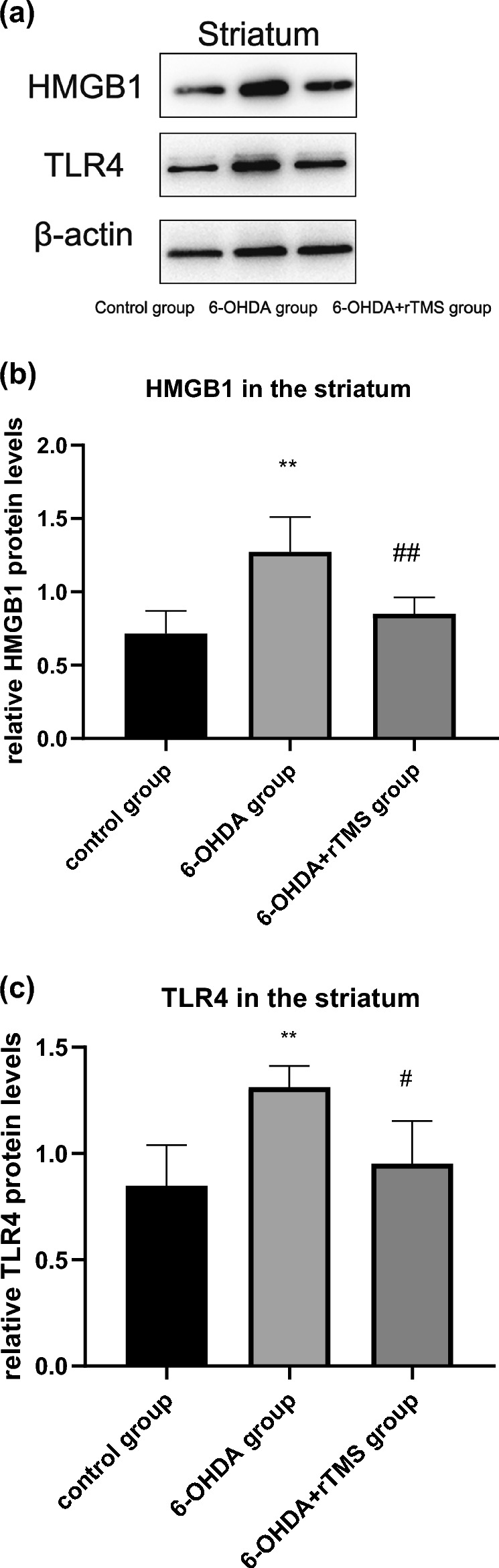
HMGB1 and TLR4 expression in the striatum assessed by Western blot analysis. **a** Typical immunoblots. **b** Mean ratio of HMGB1 densitometry density data in relation to β-actin in the striatum (∗∗P < 0.01 vs. control group, ##P < 0.01 vs. 6-OHDA group). **c** Mean ratio of TLR4 densitometry density data in relation to β-actin in the striatum (∗∗P < 0.01 vs. control group, #P < 0.05 vs. 6-OHDA group) (n = 5)

**Fig. 10:**
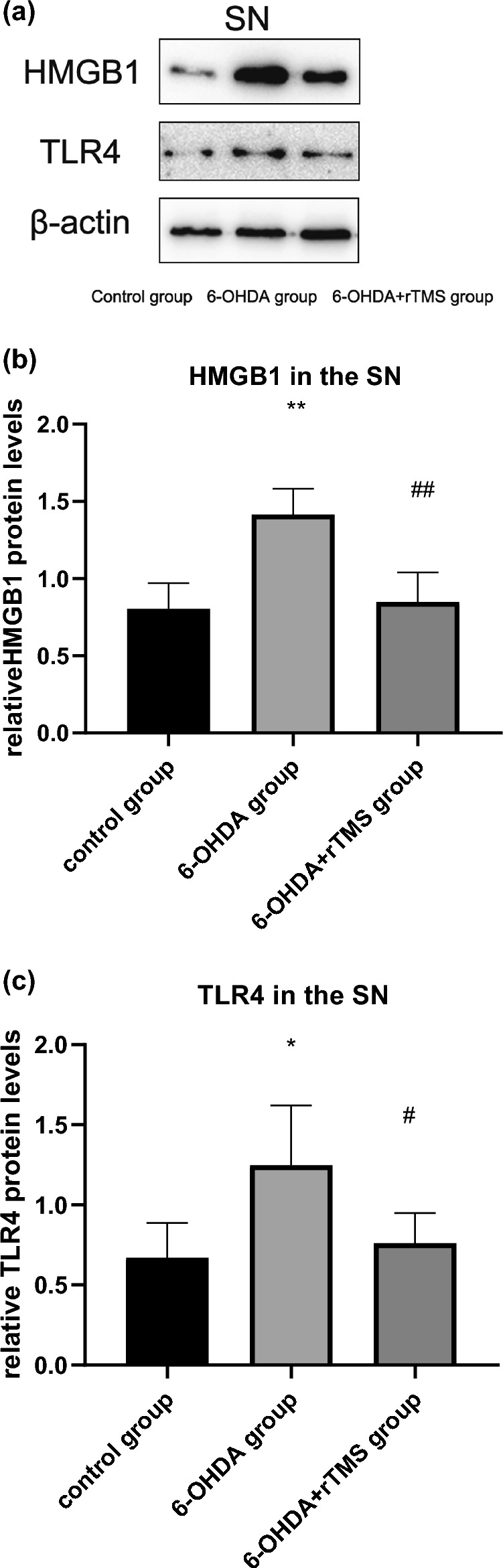
HMGB1 and TLR4 expression in the SN assessed by Western blot analysis. **a** Typical immunoblots. **b** Mean ratio of HMGB1 densitometry density data in relation to β-actin in the SN (∗∗P < 0.01 vs. control group, ##P < 0.01 vs. 6-OHDA group). **c** Mean ratio of TLR4 densitometry density data in relation to β-actin in the SN (∗P < 0.05 vs. control group, #P < 0.05 vs. 6-OHDA group) (n = 5)

In the striatum and SN, the HMGB1 data showed increased expression in the 6-OHDA group compared with the control group (P < 0.01) and decreased expression in the 6-OHDA+rTMS group compared with the 6-OHDA group (P < 0.01), as shown in Figs. 9b and 10b. For TLR4 in the striatum and SN, the 6-OHDA group showed a higher expression than the control group (P < 0.01 and P < 0.05, respectively) and the 6-OHDA+rTMS group showed a reduction in expression compared with the 6-OHDA group (P < 0.05), as shown in Figs. 9c and 10c.

## Discussion

rTMS therapy is a noninvasive, well-tolerated technique for modulating the cortical network. Previous meta-analysis studies have shown the therapeutic benefits of rTMS for PD patients [9, 10]. However, its therapeutic mechanisms still need to be explained. In this article, we explored the effects of rTMS on glia-mediated neuroinflammatory responses and the HMGB1/TLR4 pathway in PD model rats. Our study indicated that rTMS treatment ameliorated forelimb use asymmetry in PD rats and suppressed the activation of glia cells and the HMGB1/TLR4 signaling pathway.

rTMS therapy is considered to offer a high or low frequency of stimulation with distinct effects. The beneficial effects on improving motor function were reported although the optimal rTMS treatment protocol has not been determined in PD [17, 18]. A study on rTMS in PD model rats showed that after 10 Hz rTMS treatment for 4 weeks, the amphetamine-induced rotation number was significantly lower and that TH-positive DA neurons were increased in both the ipsilateral striatum and SNc [8]. They concluded that the neuroprotective effect of rTMS treatment on PD model rats might be induced by upregulating neurotrophic and growth factors [8]. In our study, after rTMS treatment, the cylinder test scores decreased significantly, and the expression level of TH was significantly increased compared with that in the 6-OHDA group. These data are in line with previous studies.

Studies from PD animal models and human postmortems have reported that neuroinflammation is important in the PD process [2]. The inflammatory response mediated by astrocytes and microglia is involved in the occurrence and development of PD [2, 4]. 6-OHDA cannot cross the blood-brain barrier [19]. It is structurally similar to dopamine and has a high affinity for dopamine transporters, which induces degeneration of dopamine neurons [19]. Oxidative stress and neuroinflammation are involved in this process [20]. In previous studies, the administration of 6-OHDA in the striatum led to increased GFAP-positive cells in both the striatum and SN [21], and glia-mediated inflammation could injure TH-positive neurons [22]. Therefore, glial activation might play an important role in TH-positive neurons lost in addition to direct impairment of 6-OHDA [23]. Since the inflammatory response is closely associated with the PD process, it is considered to be an important treatment target for PD that can alleviate glial activation and neuroinflammatory responses. In this study, astrocyte and microglia activation was observed in the 6-OHDA group, while rTMS alleviated this change.

In our study, the data showed higher expression of HMGB1 and TLR4 in the 6-OHDA group and decreased expression in the 6-OHDA+rTMS group in the striatum and SN. Angelopoulou E et al. [24] indicated that HMGB1 might play a key role in PD pathogenesis. HMGB1 has been reported to participate in neuroinflammation, autophagy, apoptosis and gene transcription regulation [24]. Neurons secreted HMGB1 in the acute stage after unilateral intrastriatal 6-OHDA infusion, whereas increased astrocytes secreted HMGB1 at the later stage [12]. After HMGB1 is secreted, it binds to its receptors, RAGE or TLRs, and the downstream signaling pathway is activated [25]. TLR2/4 receptors are expressed in the brain constitutively and can regulate inflammatory responses [26] while RAGE is usually induced by increased substrate abundance or oxidative stress [27]. Activated astrocytes and microglia accelerate HMBG1/TLR4 expression through autocrine or paracrine signaling [14], and HMGB1 promotes the activation of glial cells [24]. A study found that compared with healthy volunteers, the expression of HMGB1 and its downstream proteins was increased in PD patients [14]. rTMS may inhibit this inflammatory process. According to the results of our study, we speculate that rTMS may alleviate PD symptoms by inhibiting high glial activation and HMGB1/TLR4 expression. Downregulating the HMGB1/TLR4 signaling pathway may be beneficial for PD treatment.

## Conclusion

In conclusion, the results of our study suggested that rTMS might be a promising method for alleviating neuroinflammation by downregulating the HMGB1/TLR4 inflammatory pathway in PD rat models. Our results support the therapeutic effects of rTMS, and deepen the understanding of the mechanisms of rTMS treatment on PD.

## Data Availability

The data generated during the current study are available from the corresponding author on reasonable request.

## Abbreviations

PD: Parkinson’s disease
SN: Substantia nigra
rTMS: Repetitive transcranial magnetic stimulation
HMGB1/TLR4: High-mobility group box 1/toll-like receptor 4
RAGE: Receptor for advanced glycation end products
6-OHDA: 6-hydroxydopamine
GFAP: Glial fibrillary acidic protein
Iba-1: Ionized calcium-binding adapter molecule 1
TH: Tyrosine hydroxylase

## References

[1] Simon DK, Tanner CM, Brundin P (2020). Parkinson disease epidemiology, pathology, genetics, and pathophysiology. Clin Geriatr Med.

[2] Lee Y, Lee S, Chang SC, Lee J (2019). Significant roles of neuroinflammation in Parkinson’s disease: therapeutic targets for PD prevention. Arch Pharm Res.

[3] Zong X, Gu J, Geng D, Gao D (2022). Repetitive transcranial magnetic stimulation (rTMS) for multiple neurological conditions in rodent animal models: a systematic review. Neurochem Int.

[4] Pal R, Tiwari PC, Nath R, Pant KK (2016). Role of neuroinflammation and latent transcription factors in pathogenesis of Parkinson’s disease. Neurol Res.

[5] Troncoso-Escudero P, Parra A, Nassif M, Vidal RL (2018). Outside in: unraveling the role of neuroinflammation in the progression of Parkinson’s Disease. Front Neurol.

[6] Agostinho P, Cunha RA, Oliveira C (2010). Neuroinflammation, oxidative stress and the pathogenesis of Alzheimer’s disease. Curr Pharm Des.

[7] Harry GJ, Kraft AD (2008). Neuroinflammation and microglia: considerations and approaches for neurotoxicity assessment. Expert Opin Drug Metab Toxicol.

[8] Lee JY, Kim SH, Ko AR, Lee JS, Yu JH, Seo JH, Cho BP, Cho SR (2013) Therapeutic effects of repetitive transcranial magnetic stimulation in an animal model of Parkinson’s disease. Brain Res 6:290–302. 10.1016/j.brainres.2013.08.051 10.1016/j.brainres.2013.08.051 23998987

[9] Dong K, Zhu X, Xiao W, Gan C, Luo Y, Jiang M, Liu H, Chen X (2023). Comparative efficacy of transcranial magnetic stimulation on different targets in Parkinson’s disease: a bayesian network meta-analysis. Front Aging Neurosci.

[10] Zhang X, Jing F, Liu Y, Tang J, Hua X, Zhu J, Tuo H, Lin Q, Gao P, Liu W (2023). Effects of non-invasive brain stimulation on walking and balance ability in Parkinson’s patients: a systematic review and meta-analysis. Front Aging Neurosci.

[11] Tian Y, Chen R, Su Z (2023). HMGB1 is a potential and challenging therapeutic target for Parkinson’s Disease. Cell Mol Neurobiol.

[12] Sasaki T, Liu K, Agari T, Yasuhara T, Morimoto J, Okazaki M, Takeuchi H, Toyoshima A, Sasada S, Shinko A, Kondo A, Kameda M, Miyazaki I, Asanuma M, Borlongan CV, Nishibori M, Date I (2016). Anti-high mobility group box 1 antibody exerts neuroprotection in a rat model of Parkinson’s disease. Exp Neurol.

[13] Santoro M, Maetzler W, Stathakos P, Martin HL, Hobert MA, Rattay TW, Gasser T, Forrester IV, Berg D, Tracey KJ, Riedel G, Teismann P (2016). In-vivo evidence that high mobility group box 1 exerts deleterious effects in the 1-methyl-4-phenyl-1,2,3,6-tetrahydropyridine model and Parkinson’s disease which can be attenuated by glycyrrhizin. Neurobiol Dis.

[14] Yang Y, Han C, Guo L, Guan Q (2018). High expression of the HMGB1-TLR4 axis and its downstream signaling factors in patients with Parkinson’s disease and the relationship of pathological staging. Brain Behav.

[15] Yoon MC, Shin MS, Kim TS, Kim BK, Ko IG, Sung YH, Kim SE, Lee HH, Kim YP, Kim CJ (2007). Treadmill exercise suppresses nigrostriatal dopaminergic neuronal loss in 6-hydroxydopamine-induced Parkinson’s rats. Neurosci Lett.

[16] Ip CW, Klaus LC, Karikari AA, Visanji NP, Brotchie JM, Lang AE, Volkmann J, Koprich JB (2017). AAV1/2-induced overexpression of A53T-αsynuclein in the substantia nigra results in degeneration of the nigrostriatal system with Lewy-like pathology and motor impairment: a new mouse model for Parkinson’s disease. Acta Neuropathol Commun.

[17] Deng S, Dong Z, Pan L, Liu Y, Ye Z, Qin L, Liu Q, Qin C (2022). Effects of repetitive transcranial magnetic stimulation on gait disorders and cognitive dysfunction in Parkinson’s disease: a systematic review with meta-analysis. Brain Behav.

[18] Krogh S, Jønsson AB, Aagaard P, Kasch H (2022). Efficacy of repetitive transcranial magnetic stimulation for improving lower limb function in individuals with neurological disorders: a systematic review and meta-analysis of randomized sham-controlled trials. J Rehabil Med.

[19] Blandini F, Armentero MT (2012). Animal models of Parkinson’s disease. FEBS J.

[20] Fu Q, Song R, Yang Z, Shan Q, Chen W (2017). 6-Hydroxydopamine induces brain vascular endothelial inflammation. IUBMB Life.

[21] Hernando S, Requejo C, Herran E, Ruiz-Ortega JA, Morera-Herreras T, Lafuente JV, Ugedo L, Gainza E, Pedraz JL, Igartua M, HernandezRM (2019). Beneficial effects of n-3 polyunsaturated fatty acids administration in a partial lesion model of Parkinson’s disease: the role of glia and NRf2 regulation. Neurobiol Dis.

[22] Saijo K, Winner B, Carson CT, Collier JG, Boyer L, Rosenfeld MG, Gage FH, Glass CK (2009). A Nurr1/CoREST pathway in microglia and astrocytes protects dopaminergic neurons from inflammation-induced death. Cell.

[23] Wei R, Rong C, Xie Q, Wu S, Feng Y, Wang R, Dai Z, Lin T (2019). Neuroprotective effect of optimized Yinxieling formula in 6-OHDA-induced chronic model of Parkinson’s disease through the inflammation pathway. Evid Based Complement Alternat Med.

[24] Angelopoulou E, Piperi C, Papavassiliou AG (2018). High-mobility group box 1 in Parkinson’s disease: from pathogenesis to therapeutic approaches. J Neurochem.

[25] Mohamed YT, Salama A, Rabie MA, Abd El Fattah MA (2023). Neuroprotective effect of secukinumab against rotenone induced Parkinson’s disease in rat model: involvement of IL-17, HMGB-1/TLR4 axis and BDNF/TrKB cascade. Int Immunopharmacol.

[26] Crack PJ, Bray PJ (2007). Toll-like receptors in the brain and their potential roles in neuropathology. Immunol Cell Biol.

[27] Greco R, Amantea D, Mangione AS, Petrelli F, Gentile R, Nappi G, Blandini F, Corasaniti MT, Tassorelli C (2012). Modulation of RAGE isoforms expression in the brain and plasma of rats exposed to transient focal cerebral ischemia. Neurochem Res.

